# Charlson comorbidity index predicts the 10-year survivorship of the operatively treated hip fracture patients

**DOI:** 10.1007/s00590-022-03259-2

**Published:** 2022-04-18

**Authors:** Simo S. A. Miettinen, Susanna Savolainen, Heikki Kröger

**Affiliations:** 1 Department of Orthopaedics, Traumatology and Hand Surgery, Kuopio University Hospital, P.O. Box 1777, 70211 Kuopio, Finland; 2 Faculty of Health Sciences, University of Eastern Finland, Yliopistonranta 1, 70210 Kuopio, Finland

**Keywords:** Survival, Complications, Mortality, Outcome, Proximal femoral fractures, Femoral neck fractures, Hip fractures, Pertrochanteric fracture, Charlson comorbidity index

## Abstract

**Purpose:**

The aim of this study was to determine how Charlson comorbidity index (CCI) predicts the 10-year survival of operatively treated hip fracture patients aged ≥ 65 years.

**Methods:**

This retrospective cohort study included all consecutive patients who had a hip fracture and were operatively treated upon in the study period from 01 January 2007 to 31 December 2007 at the university hospital. The clinical patient data were obtained from the medical records, and CCI score was calculated. The CCI predicts the 10-year mortality for a patient who may have a range of 22 comorbid conditions. Cumulative survival and complications were evaluated in terms of gender.

**Results:**

A total of 241 hip fractures were studied; of these, 183/241 (76%) were females. A total of 32/241 (15%) complications were found, of which 26/241 (11%) were considered major. Overall, 213/241 (88%) patients died during the 10 years of follow-up. Cumulative survival estimates for females were 13% at 10 years (SE = 0.3, 95% CI 3.8–4.8), and for males, it was 12% at 10 years (SE = 0.5, 95% CI 2.8–4.6) (*p* = 0.33). CCI was significantly associated with mortality after the hip fracture as patients with CCI scores ≥ 4 were at a 3.1–8.5 times higher risk of death compared to patients with low CCI scores of 2–3 (*p* < 0.001).

**Conclusion:**

Complications are common after operatively treated hip fracture. Advanced age, living in a care facility, ASA class 4 and high CCI score ≥ 4 were risk factors of mortality after the operatively treated hip fracture.

## Introduction

A hip fracture is a common cause of disability and death among the elderly, and particularly in women [1]. The key factor in predicting the future incidence of hip fracture is the progressive ageing of the population in western countries [2]. Hip fractures are associated with significant mortality, particularly in patients with pre-existing comorbidities; the risk of mortality is highest in the first 4 weeks after fracture [3]. The one-year mortality of operatively treated hip fracture patients aged over 65 years varies from 16.6 to 18.7%, while the 10-year mortality varies from 75.3 to 81.5% [3–5].

Hip fracture patients often have significant comorbidity, and continuous improvements are made to optimise the treatment of this patient group. Numerous studies have shown the association of pre-fracture comorbidity with post-operative complications and mortality [6, 7]. Although predictors for mortality after hip fracture have been studied extensively, the everyday usage of risk prediction models is relatively limited. With the pre-surgical assessment of risk factors using these models and the application of prophylactic interventions, the survival of patients could be improved. One such model is the Charlson comorbidity index (CCI), which predicts the 10-year mortality for a patient who may have a range of comorbid conditions [8]. CCI has been used as a guide to predict 30-day and one-year mortality rates of the hip fracture patients, and its usefulness has been shown to be valuable [9, 10]. However, there is a lack of hip fracture studies in which CCI has been used in its originally intended time frame as the predictor of the 10-year mortality of these patients. This information is particularly vital in an ageing population with greater access to health care as the proper identification and management of higher risk patients can reduce mortality.

The aim of this study was to determine how CCI predicts the 10-year survival of operatively treated hip fracture patients aged 65 years or older. In addition, 30-days, 1-year, 2-year and 5-year survivals and complications were studied. Special study interest was reported in gender comparison in terms of survival and complications. We hypothesize that increased age, male gender and higher CCI score will be associated with a higher rate of post-operative complications and mortality during the 10-year follow-up.

## Materials and methods

### Participants and setting

A retrospective cohort study with prospective data collection was performed for all consecutive patients who had a hip fracture and were operatively treated upon in the study period from 01 Jan 2007 to 31 Dec 2007 at the University Hospital. There are approximately 245,000 inhabitants living in the area of the Hospital. Patients were screened from the hospital medical registry and sorted according to the ICD-10 (International Classification of Diseases) coding system; the codes S72.0, S72.1 and S72.2 were used. The operative coding system NCSP (Nordic Classification of Surgical Procedures) codes NFJ50, NFJ52, NFJ54, NFJ62, NFJ64, NFB10–NFB50 and NFB99 were used. Inclusion criteria were age ≥ 65 years and an acute low-energy hip fracture which was operated on. The exclusion criteria were age < 65 years, conservative treatment of the fracture, pathological fracture, periprosthetic fracture, high energy fracture and open fracture. The 10-year follow-up time ended on 31 Dec 2017 or with the death of the patient.

### Variables and outcomes

The clinical patient data were obtained from the medical records, including gender, age, body mass index (BMI), place of living, diagnosis of osteoporosis or osteopenia prior to the hip fracture, functionally impaired movement capacity (due to alcohol abuse or neurological disorder), hospitalisation time, types of fracture, injury side, time from injury to surgery, The American Society of Anaesthesiologists (ASA) classification and Charlson comorbidity index (CCI). The CCI predicts the 10-year mortality for any patient who may have a range of 22 comorbid conditions, such as heart disease, AIDS, or cancer [8]. Each comorbidity category has an associated weight (from 1 to 6), based on the adjusted risk of mortality or resource use, and the sum of all weights provides a single comorbidity score for each patient. A score of zero indicates that no comorbidities were found and a patient with a CCI score between 1–2 and 3–4 had mortality rates (after 1-year follow-up) of 26% and 52% [8]. The higher the score, the more likely that the predicted outcome will result in mortality or higher resource use [8]. The age groups 65–79 years, 80–90 years and > 90 years were formed to more precisely evaluate the effect of age on mortality. The CCI was chosen as it has been demonstrated as one of the most predictive scores of mortality after the hip fracture, even though it is not initially designed for hip fracture patients [9, 10].

The types of complications were evaluated and further classified as major and minor types. Major complications were defined as a need for revision surgery for any reason, like loosening of the implant, dislocation, avascular osteonecrosis, non-union and broken implants. The other major adverse events were cardiac attack, pulmonary embolus or deep venous thrombosis and death of the patient related to the hip fracture. Minor complications were defined non-life-threatening complications like superficial infection and pain problems for unknown reasons.

### Ethics

All procedures performed in studies involving human participants were in accordance with the ethical standards of the institutional and/or national research committee and with the 1964 Helsinki declaration and its later amendments or comparable ethical standards. The ethical review committee of the Hospital gave permission (892/13.02.00/2019) for this study. The study protocol and evaluation of patient medical records were approved by the Organisational Board of the Hospital (172/2019).

### Statistical analysis

Kaplan–Meier and log-rank tests were used to study post-operative survival at 30 days, 1 year, 2 years, 5 years and 10 years. Comparisons of continuous data were performed using the Mann–Whitney U test. For categorical data, a chi-square test was used. The independent-sample t-test was used for parametric data comparisons. Cox regression models were used for both univariate (single risk factors) and multivariate (combinations of risk factors) analyses to evaluate the most common risk factors for death and complications according to the previous literature [6–8]. These risk factors were age, gender, waiting time to hip fracture operation from the hip fracture, place of living (home or care facility), ASA-classification, operation diagnosis (S72.0, S72.1 and S72.2), operative code (NFB10-99 and NFJ50-64), surgeon (resident or consultant) and CCI. The univariate analysis was followed by multivariate analysis to adjust for other independent factors, including confounders. Risk ratio along with 95% CI was reported for each variable that was significant in both uni- and multivariate analysis. The CCI scores were divided to 4 groups [I: 2–3, II: 4, III: 5–6 and IV: ≥ 7] for between group comparisons. All *p* values < 0.05 were considered statistically significant. Data were analysed using SPSS (SPSS Inc., Chicago, IL, USA. Ver 27.0.0, IBM).

## Results

A total of 241 operatively treated hip fractures met the inclusion criteria for this study. The mean age of the patients was 81.4 (SD 6.8, range 65.0–99.0) years, and most of the participants were female (183/241; 76%). The mean age of females was 82.3 (SD 6.9, range 65.0–99.0) years and of males was 78.7 (SD 5.8, range 65.0–93.0) years (*p* < 0.001). The right side was operated on in 133/241 (55%) hip fractures. The mean waiting time to operation from hip fracture was 4.0 (SD 12.0, range 0–108) days, and the mean inpatient time was 3.3 (SD 23.6, range 1–51) days. The mean follow-up time of females was 3.4 years (SD 3.4, range 1 day–10.8 years) and of males was 3.3 years (SD 3.3, range 1 day–10.6 years) (*p* = 0.17). The mean CCI-score of females was 4.8 (SD 1.5, range 2–10) and of males was 4.9 (SD 1.7, range 2–11) (*p* = 0.69). Demographic baseline characteristics of the patients in terms of gender are given in more detail in Table 1.

**Table 1:** Demographic characteristics, personal history, comorbidities and treatment in patients with hip fractures in terms of gender

	Females	Males	*p*-value
	*n* (%)	*n* (%)	
*Age groups*			0.005^a^
65–79 years	61 (33)	29 (50)	
80–90 years	105 (57)	28 (48)	
> 90 years	17 (9)	1 (2)	
*Comorbidities*			
Osteoporosis or osteopenia	35 (19)	2 (3)	0.004^b^
Neurological disease	55 (30)	22 (38)	0.262^b^
Alcoholism	2 (1)	5 (9)	0.003^b^
None of the above	91 (50)	34 (50)	
*Prefracture place of living*			0.017^b^
Home	132 (72)	32 (55)	
Care facility	49 (27)	25 (43)	
Unknown	2 (1)	1 (2)	
*ASA-classification*			0.120^c^
II	5 (9)	–	
III	32 (59)	20 (80)	
IV	17 (32)	5 (20)	
Unknown	129	33	
*Operation diagnosis*			0.297^c^
S72.0 Collum fracture	103 (56)	39 (67)	
S72.1 Perthrochanteric fracture	63 (34)	16 (28)	
S72.2 Subthrochanteric fracture	17 (9)	3 (5)	

^a^Mann-Whitney *U* test

^b^Independent-sample *t* test

^c^A Chi-square test

**Fig. 1:**
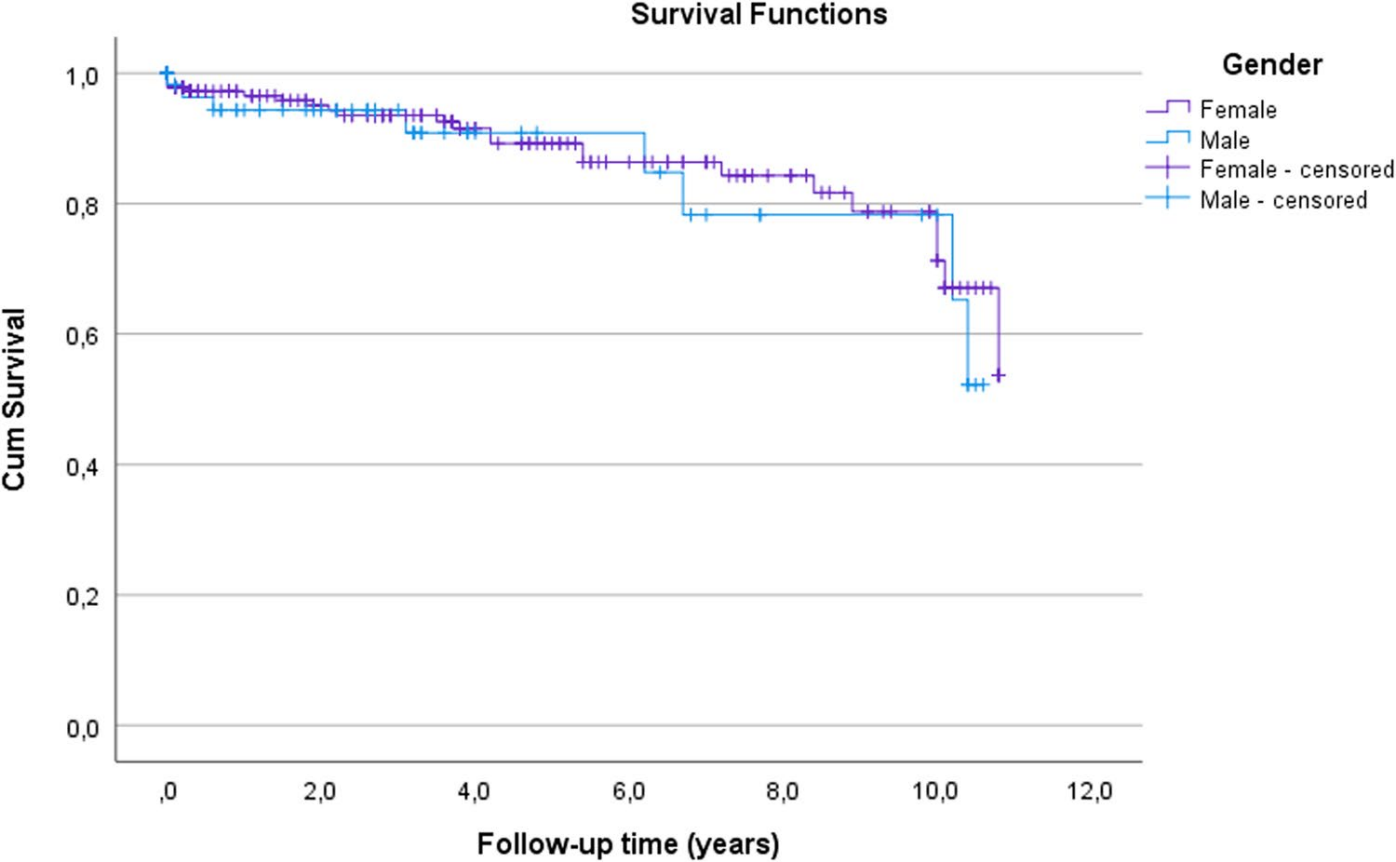
Kaplan–Meier survival analysis of time to revision surgery due to major adverse event in terms of gender. Cumulative survival estimates without any major adverse event for females were 98% at 30 days, 97% at 1 year, 95% at 2 years, 89% at 5 years, 71% at 10 years and 54% at 10.8 years (SE = 0.3, CI 95% 8.8–10.0); for males, these estimates were 98% at 30 days, 94% at 1 year, 94% at 2 year, 91% at 5 years, 78% at 10 years and 52% at 10 years (SE = 0.5, CI 95% 8.2–10.2). Log-rank test, *p* = 0.61

A total of 32/241 (15%) complications were found; of these, 31/241 (13%) were considered major adverse events, with 16/31 (52%) requiring operative treatment (Table 2). The mean time to any complication was 106 (SD 253, range 0–1042) days. Cumulative survival estimates without any major adverse event in terms of gender are shown in Fig. 1. The univariate and multivariate Cox regression models did not show any statistically significant risk factors for complication (data not shown). There was no statistically significant difference between CCI groups in terms of major complications (*p* = n.s.).

**Table 2:** Complications and mortality in terms of gender

	Females	Males	*p*-value
	*n* (%)	*n* (%)	
*Complication*			0.62^a^
None	166 (88)	59 (87)	
Yes	23 (12)	9 (13)	
Major	19 (11)	7 (10)	
Periprosthetic fracture	4 (2)	–	
Cardiac complication	4 (2)	1 (2)	
Conversion of SEP to TEP	2 (1)	1 (2)	
Non-union of the fracture	2 (1)	2 (3)	
Infection, deep	1 (0.5)	1 (2)	
Removal of the SEP due to recurrent dislocation	1 (0.5)	1 (2)	
Dislocation	1 (0.5)	–	
Loosening of the implant	1 (0.5)	–	
Posttraumatic osteoarthrosis	1 (0.5)	–	
Post operative haemorrghage	1 (0.5)	–	
Exchange of gammanail to SEP	1 (0.5)	–	
Other	–	1 (2)	
Minor	4 (2)	2 (3)	
Infection, superficial	3 (2)	1 (2)	
Pneumonia	1 (0.5)	–	
Decubitus	–	1 (2)	
*Mortality*			
30-day mortality	9 (5)	8 (14)	0.02^b^
1-year mortality	42 (23)	16 (28)	0.47^b^
2-year mortality	59 (32)	22 (38)	0.42^b^
5-year mortality	115 (63)	43 (74)	0.12^b^
10-year mortality	162 (89)	51 (88)	0.90^b^

*SEP* semiendoprosthesis, *TEP* total endoprosthesis

^a^Independent-sample *t* test

^b^Mann-Whitney *U* test

**Fig. 2:**
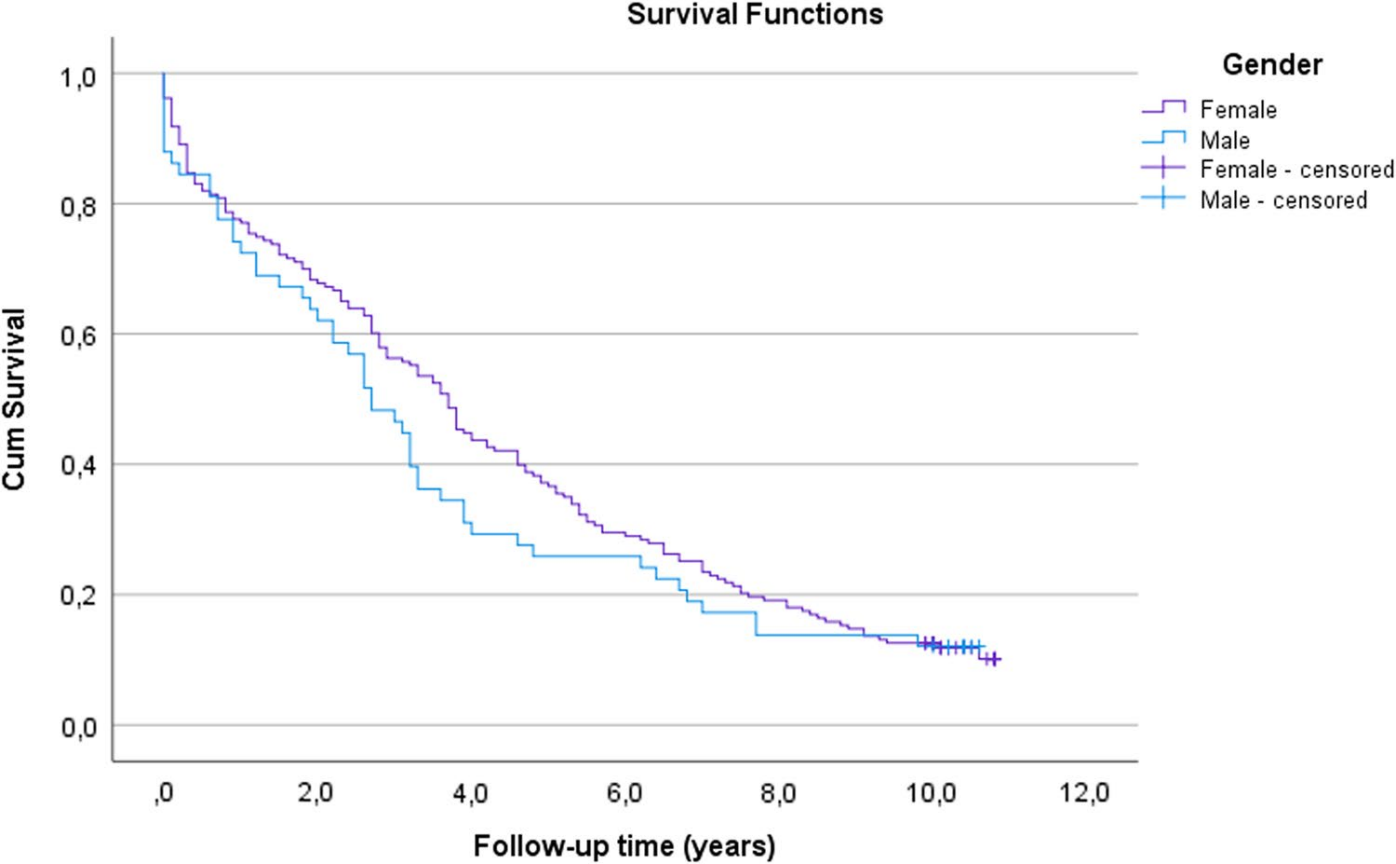
Kaplan–Meier survival analysis of time to death after the operatively treated hip fracture. Cumulative survival estimates for females were 96% at 30 days, 77% at 1 year, 68% at 2 years, 37% at 5 years and 13% at 10 years (SE = 0.3, CI 95% 3.8–4.8); for males, these estimates were 86% at 30 days, 72% at 1 year, 62% at 2 year, 26% at 5 years and 12% at 10 years (SE = 0.5, CI 95% 2.8–4.6). Log-rank test, *p* = 0.33

A total of 213/241 (88%) patients died during the 10-year follow-up. The mortality of hip fracture patients during the first 30 days was statistically higher in males compared with females (Table 2). Cumulative survival estimates to death after the operatively treated hip fracture in terms of gender are shown in Fig. 2.

**Fig. 3:**
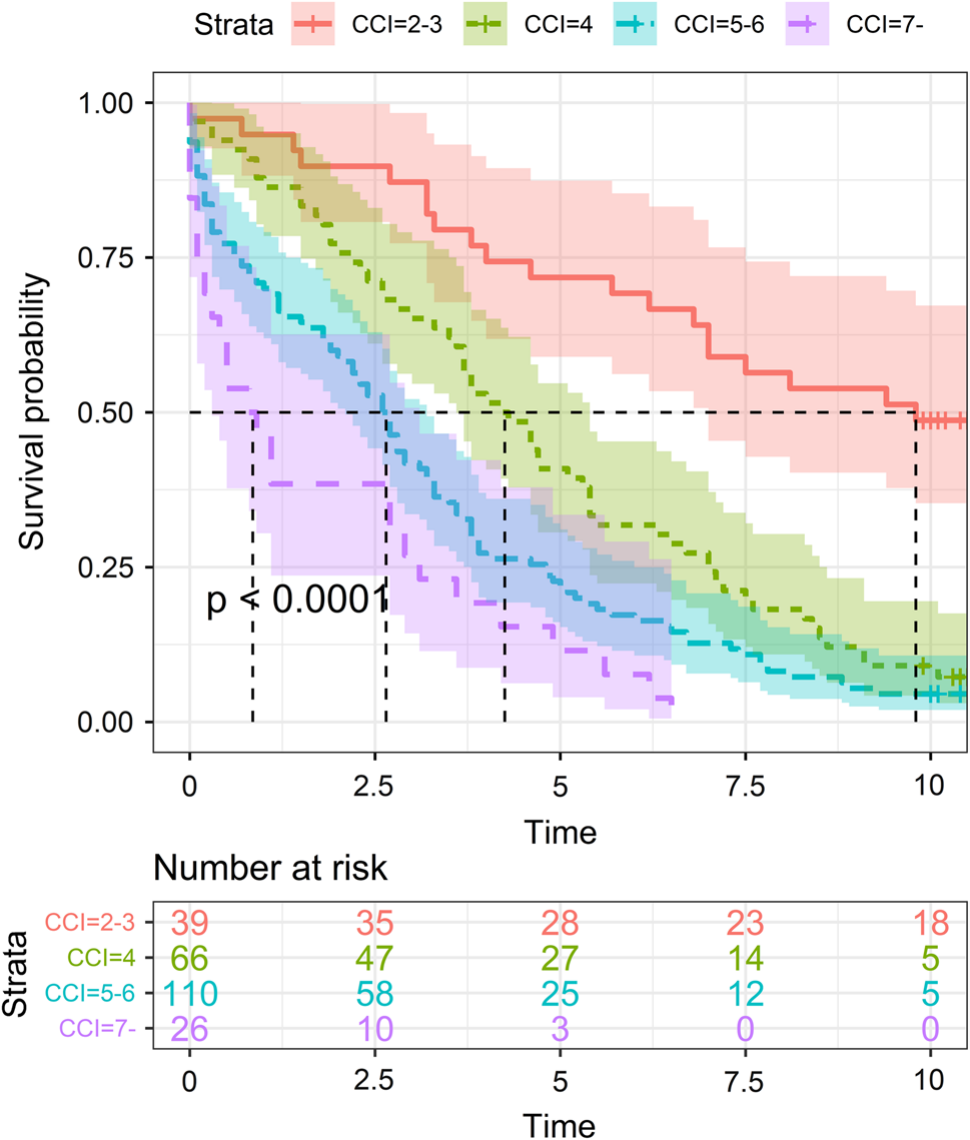
The median survival time in terms of Charlson Comorbidity Index (CCI) score groups [I) 2–3, II) 4, III 5–6 and IV) ≥ 7]. In group I, the median survival time was 9.8 (95% CI 7.0–Inf.) years, in group II 4.3 (95% CI 3.6–5.4) years, in group III 2.7 (95% CI 2.1–3.2) years and in group IV 0.9 (95% CI 0.3–3.1) years (*p* < 0.001)

The median survival time in CCI score group IV was statistically significantly lower than in groups I-III (Fig. 3). The univariate and multivariate Cox regression models showed that age, ASA level 4 and living in a care facility were significant risk factors for death after hip fracture (Table 3). CCI was also significantly associated with mortality after hip fracture, as patients with CCI scores ≥ 4 were at 3.1–8.5 times higher risk of death compared to patients with low CCI scores of 2–3 (Table 3).

**Table 3:** The preoperative risk factors for death after the hip fracture. The Cox regression univariate (single risk factors) and multivariate (combinations of risk factors) model

	Univariate			Multivariate		
	HR	95% CI	*p*-value	HR	95% CI	*p*-value
Age	1.08	(1.06–1.11)	< 0.001	1.05	(1.03–1.08)	< 0.001
Gender						
Female	1					
Male	1.17	(0.85–1.60)	0.33			
ASA-classification						
2	1					
3	1.9	(0.68–5.28)	0.22			
4	3.16	(1.08–9.23)	0.04			
Prefracture place of living						
Home	1			1		
Care facility	2.27	(1.69–3.03)	< 0.001	1.71	(1.26–2.31)	< 0.001
Waiting time (days) to operation from the fracture	1	(0.99–1.01)	0.64			
Operation diagnosis						
S72.0 Collum fracture	1					
S72.1 Perthrochanteric fracture	1.11	(0.83–1.48)	0.49			
S72.2 Subthrochanteric fracture	0.69	(0.41–1.19)	0.18			
Operative codes						
NFB10-99 Arthroplasty	1					
NFJ50-64 Open reduction and internal fixation	0.88	(0.67–1.15)	0.36			
Surgeon						
Resident	1					
Consultant	1.01	(0.75–1.39)	0.88			
CCI	1.38	(1.27–1.49)	< 0.001			
CCI (ref = 2–3)						
4	3.13	(1.88–5.22)	< 0.001	2	(1.16–3.47)	0.013
5–6	4.78	(2.93–7.79)	< 0.001	2.58	(1.47–4.51)	< 0.001
7–	8.52	(4.65–15.59)	< 0.001	4.46	(2.3–8.67)	< 0.001

## Discussion

This study showed that there is a significant association between CCI score and the mortality of hip fracture patients during the 10-year follow-up. Patients with a high CCI score ≥ 4 had a 3.1–8.5 times higher risk of death during the follow-up compared to patients with lower CCI scores. This study finding supports previous studies where a CCI ≥ 4 has been associated with a higher mortality rate [10, 11].

The survival of hip fracture patients in this study is consistent with the findings of several other similar studies where high rates of mortality have been shown in patients aged > 65 years [10–14]. The 30-day mortality of hip fracture patients aged > 65 years varied from 6.4 to 7.5%, which is similar to the findings of this study [12, 15]. The 1-year mortality varied from 11.9 to 25.6%, and the 2-year mortality varied from 18.5 to 32.5% in previous studies, which are in line with the results of this study [3–5, 12]. The 5-year mortality rates of this study are similar to previously published studies, with rates varying from 55 to 61.5% [5, 16]. The 10-year mortality rates of previous studies are parallel to those of this study, varying from 75.3 to 81.5% [3–5]. Interestingly, in this study, the mean age of the male hip fracture patients was 78.7 years, which is higher than general life expectancy 75.8 years of the males was in our hospital district at year 2007 [17]. In contrast, the life expectancy of the females in 2007 was 82.9 years in our hospital district, while in our study, the mean age of female hip fracture patients was 82.3 years [17].

Besides age and CCI, this study also revealed other factors that correlated with mortality. The risk of death after hip fracture was highest during the first 30 days in males, but the overall survival of males in the 65–79 year age group was higher than in females of the same age group during the first 2 post-operative years; this difference reversed after the 2-year follow-up. In older age groups, females had a lower risk of death from the start of the follow-up. Parallel findings have been shown previously, as males in general have been shown to be at a higher risk of death compared to females [4, 12, 18]. However, in this study, Cox regression showed that male gender was not a statistically significant risk factor for death after hip fracture, and a similar disparity in mortality between the genders has been reported previously [19]. In a recent literature review, it was found that age at admission with a hip fracture depends on the patient’s residence and gender, as it was common for men and those admitted from long-term care to be older than women and those admitted from private homes [20]. Moreover, it has been shown that the presence of chronic conditions depends on the patient’s gender and residence, with men and those admitted from long-term care typically presenting with more chronic conditions than women and those admitted from home [20]. The results of this study are consistent with previous findings, as we found that males were younger than females and more commonly lived in care facilities [21, 22]. In this study, men tend to be in a worse prehospital condition, as their ASA-classification was inferior to that of females and ASA was found to be a statistically significant risk factor for death. Similar findings have been recently shown by Haugan et al., who found that ASA predicts the 30-day mortality of hip fracture patients [12]. In general, it has been confirmed that absolute and relative mortality rates of hip fracture patients increase with ASA physical status [22].

Operation diagnosis, fracture type, surgeon or waiting time to operation after the hip fracture were not risk factors for death in this study. There is a consensus which indicates that surgery for hip fracture should be performed within 24 h of injury to reduce the risk of post-operative complications and mortality [23]. However, there is some debate about whether surgery should be delayed among patients who are medically unstable at admission to provide the opportunity to optimise patients’ medical status and thereby decrease risk of perioperative complications [20, 24]. The results of this study do not support previous findings, where operation diagnosis, fracture type and type of surgery have been associated with a higher risk of death after hip fracture [20]. This study did not find surgeon to be a risk factor for death, which supports previous findings where outcomes for trainees performing arthroplasty for hip fracture are equivalent to those of consultant surgeons [25].

In this study, the overall complication rate was 15%, and the most common complication was periprosthetic fracture. Similar findings have been shown previously by Cher et al., who found that the rate of post-operative complications is high and is linked to advanced age and comorbidities of hip fracture patients [11]. Other common complications in this study were related to cardiac morbidities and periprosthetic fractures, the rates of which were parallel to previous studies [5, 26].

This retrospective study design had some inherent limitations, which could have been minimised by a prospective study design. The typical retrospective study flaws also limited our study; for example, researchers were not able to see the patients and had to rely on patients’ medical records and it is possible that more comorbidity was present than recorded. In addition, no clinical evaluation could be performed, and there were no patient-reported outcome measurements available. For these reasons, the endpoint was the death of the patient. Moreover, we were not able to control for other potential confounding factors that might have affected our results, such as other comorbid conditions not accounted for by CCI, medications, smoking status of the patients, surgeon experience and post-operative care of the patient by caregivers. Apart from that, data were collected from a single institution, which might not be representative of the whole country.

## Conclusions

Complications are common after operatively treated hip fracture. Increasing age, living in a care facility, ASA class 4 and high CCI score ≥ 4 were risk factors for mortality after operatively treated hip fracture. Advanced age > 80 years and male gender predict poor 10-year survival after operatively treated hip fracture.
